# Supplementary material for: Impaired Proteostasis is an Early Feature of the Diabetic Heart in Humans and Mice
Source: bioRxiv. 2026 Sep 2:2026.09.01.748593. Preprint. [Version 1] doi: 10.64898/2026.09.01.748593 (PMC13684640; doi:10.64898/2026.09.01.748593)
Supplement: Supplementary file 1 [file media-1.pdf]

Supplementary Figures and Tables

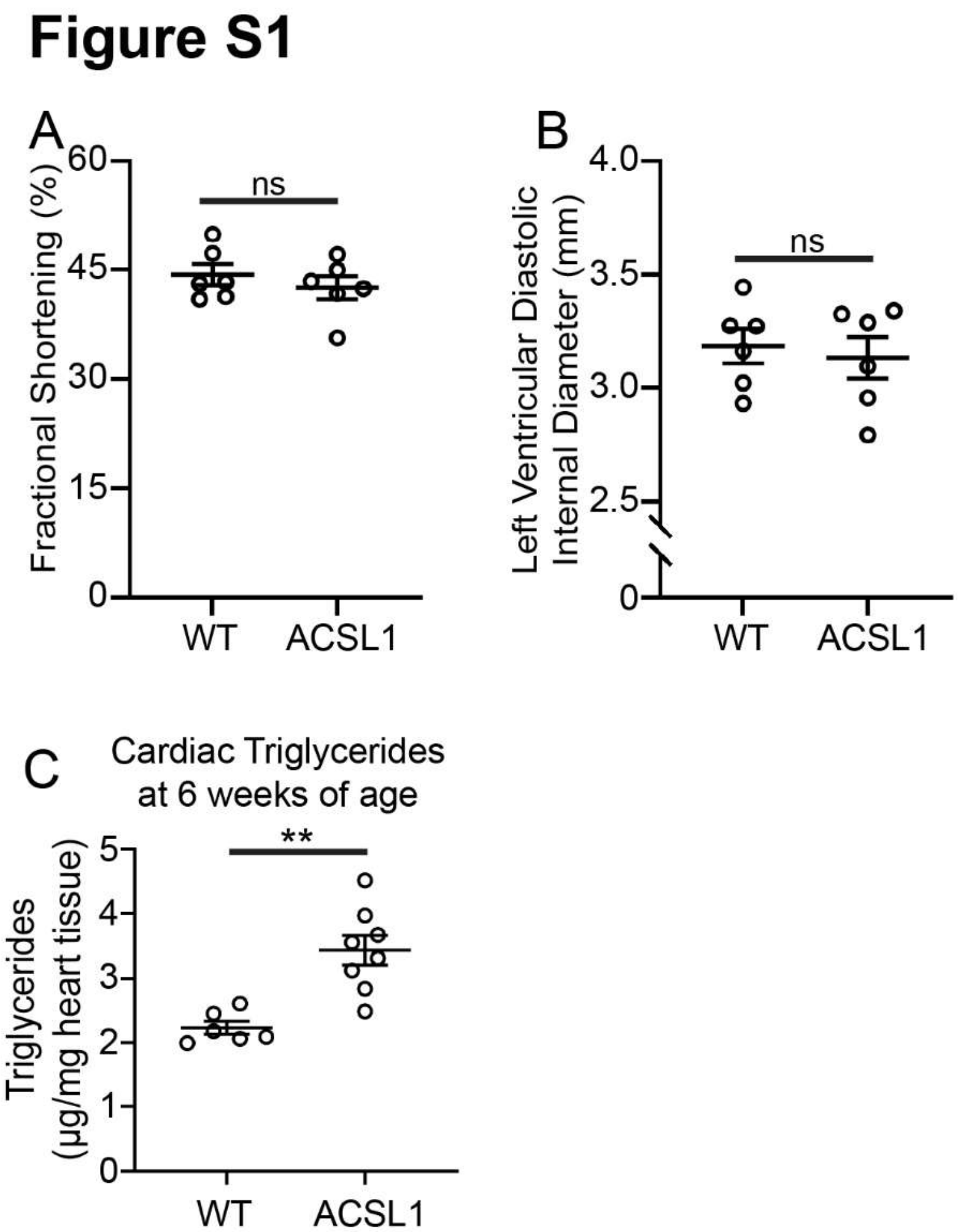

Supplemental Figure S1. Six-week-old ACSL1 hearts exhibit normal cardiac function by echocardiography and increased triglyceride levels. (A-B) Quantitation of left ventricular endocardial fractional shortening (A) and left ventricular diastolic internal diameter (B) in wild-type (WT) or MHC-ACSL1 transgenic mice (ACSL1) at 6 weeks of age. Statistical testing by t-test, ns $p>0.05$. **(C)** Triglyceride levels in 6-week-old wild-type (WT) and MHC-ACSL1 transgenic mouse hearts. Statistical testing by t-test, ** $p<0.01$.

Figure S2

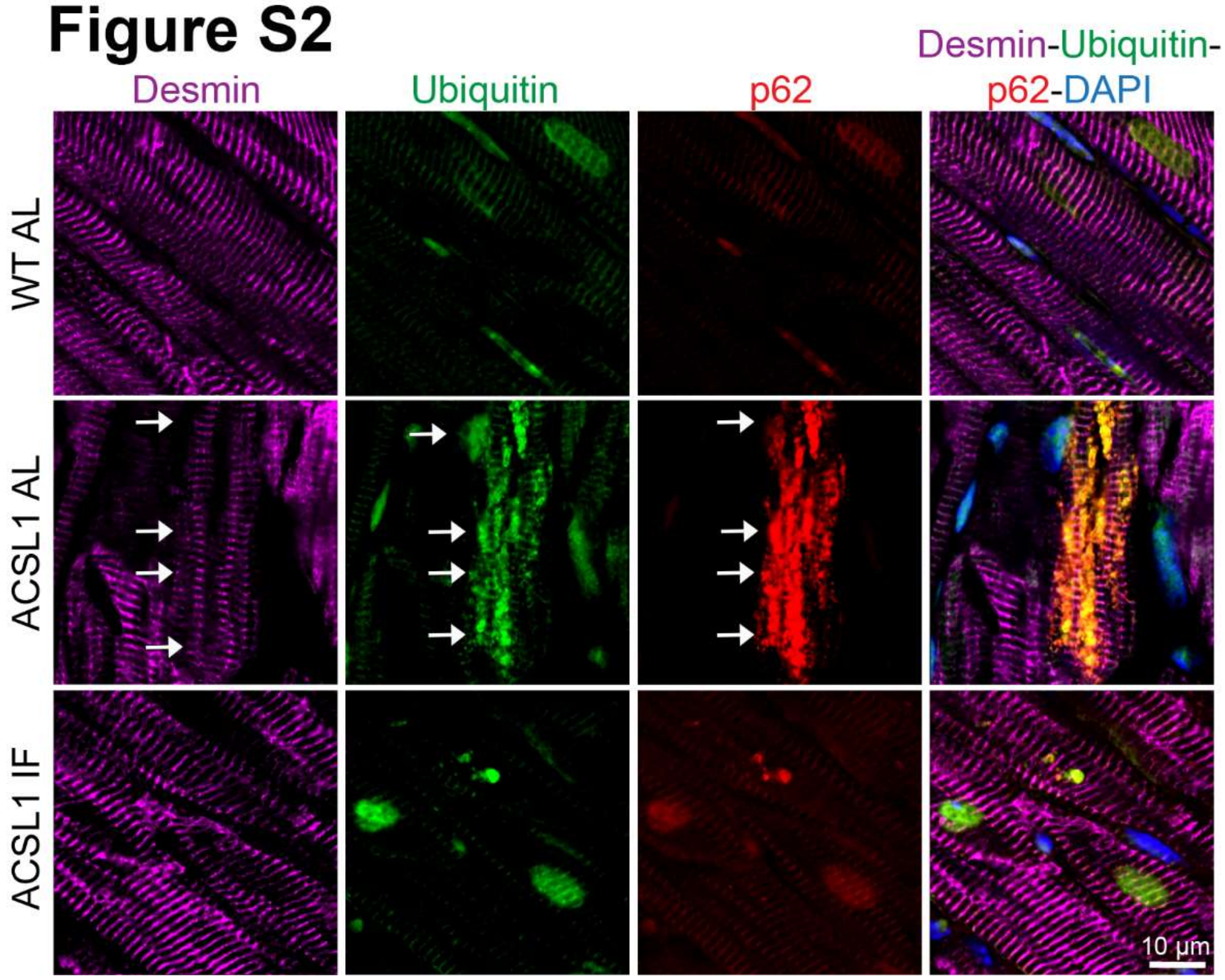

Supplemental Figure S2. Intermittent fasting rescues protein aggregate pathology in the myocardium. (A) Immunohistochemistry staining of 12-week-old hearts from wild-type (WT) or MHC-ACSL1 transgenic hearts under ad-libitum (AL) or intermittent fasting (IF) dietary treatment. Images show staining for Desmin (magenta), ubiquitin (green), p62 (red), and DAPI (blue). Arrows point to protein aggregates.

Figure S3

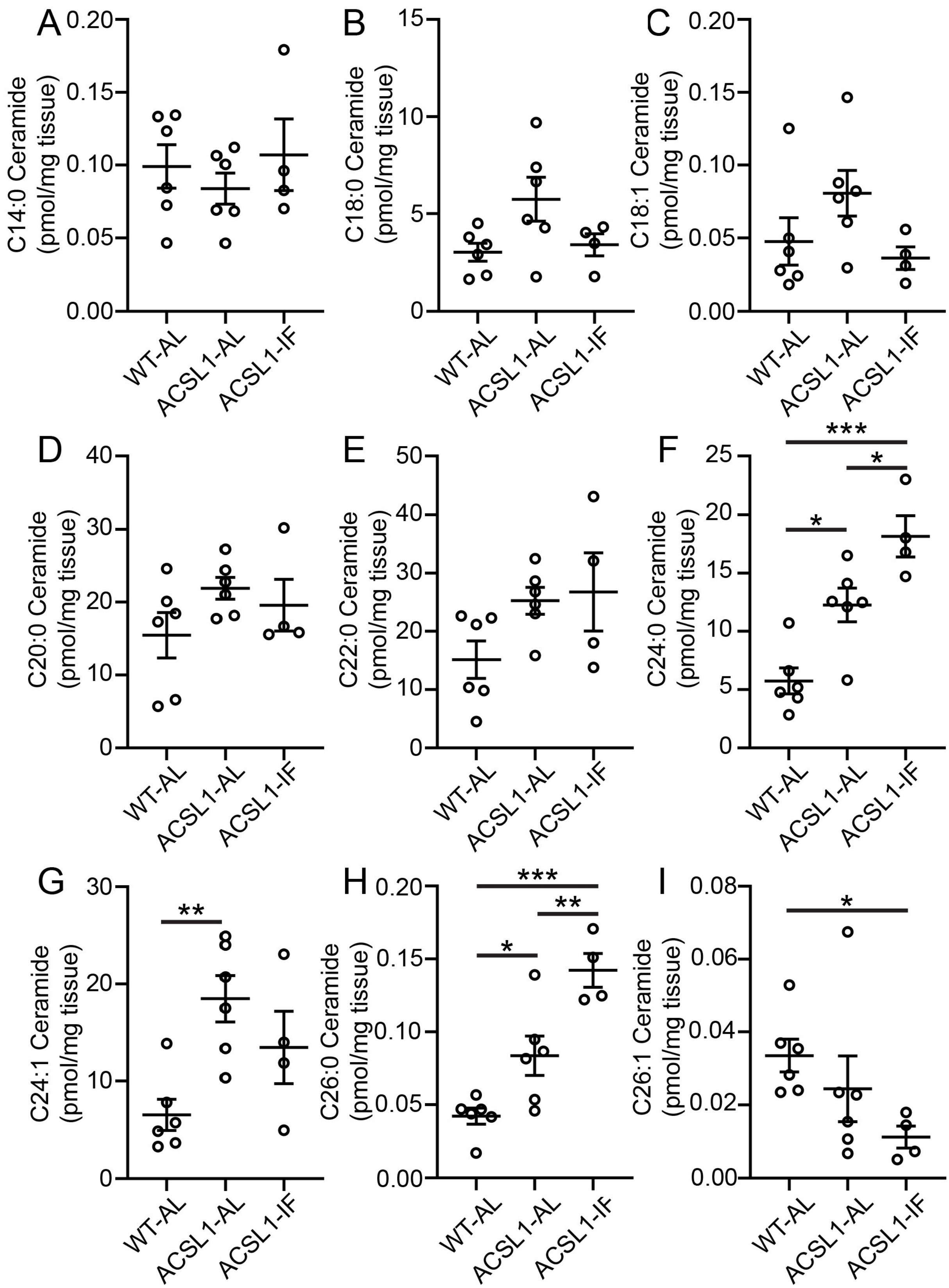
A
C14:0 Ceramide (pmol/mg tissue)
0.00
0.05
0.10
0.15
0.20
WT-AL
ACSL1-AL
ACSL1-IF
B
C18:0 Ceramide (pmol/mg tissue)
0
5
10
15
C
C18:1 Ceramide (pmol/mg tissue)
D
C20:0 Ceramide (pmol/mg tissue)
0
10
20
30
40
E
C22:0 Ceramide (pmol/mg tissue)
50
F
C24:0 Ceramide (pmol/mg tissue)
25
***
*
*
G
C24:1 Ceramide (pmol/mg tissue)
**
H
C26:0 Ceramide (pmol/mg tissue)
***
**
*
I
C26:1 Ceramide (pmol/mg tissue)
0.00
0.02
0.04
0.06
0.08
*

Supplemental Figure S3. Effects of cardiac lipid overload in ACSL1 mouse hearts under ad-lib and intermittent fasting conditions. (A-I) Levels of indicated ceramide species in 12-week-old mouse hearts from wild-type mice on ad-libitum diet (WT AL), ACSL1 transgenic mice on ad-libitum diet (ACSL1 AL), or ACSL1 transgenic mice after 6 weeks of every-other-day intermittent fasting (ACSL1 IF) from age 6 weeks to 12 weeks. Ceramide levels are normalized to tissue mass. Statistical testing is by one-way ANOVA followed by post-hoc testing by Tukey's test (A, B, E, F, G, H) or by Kruskal-Wallis test with post-hoc testing by Dunn's test (C, D, I). * $p < 0.05$, ** $p<0.01$, *** $P<0.001$.

Figure S4

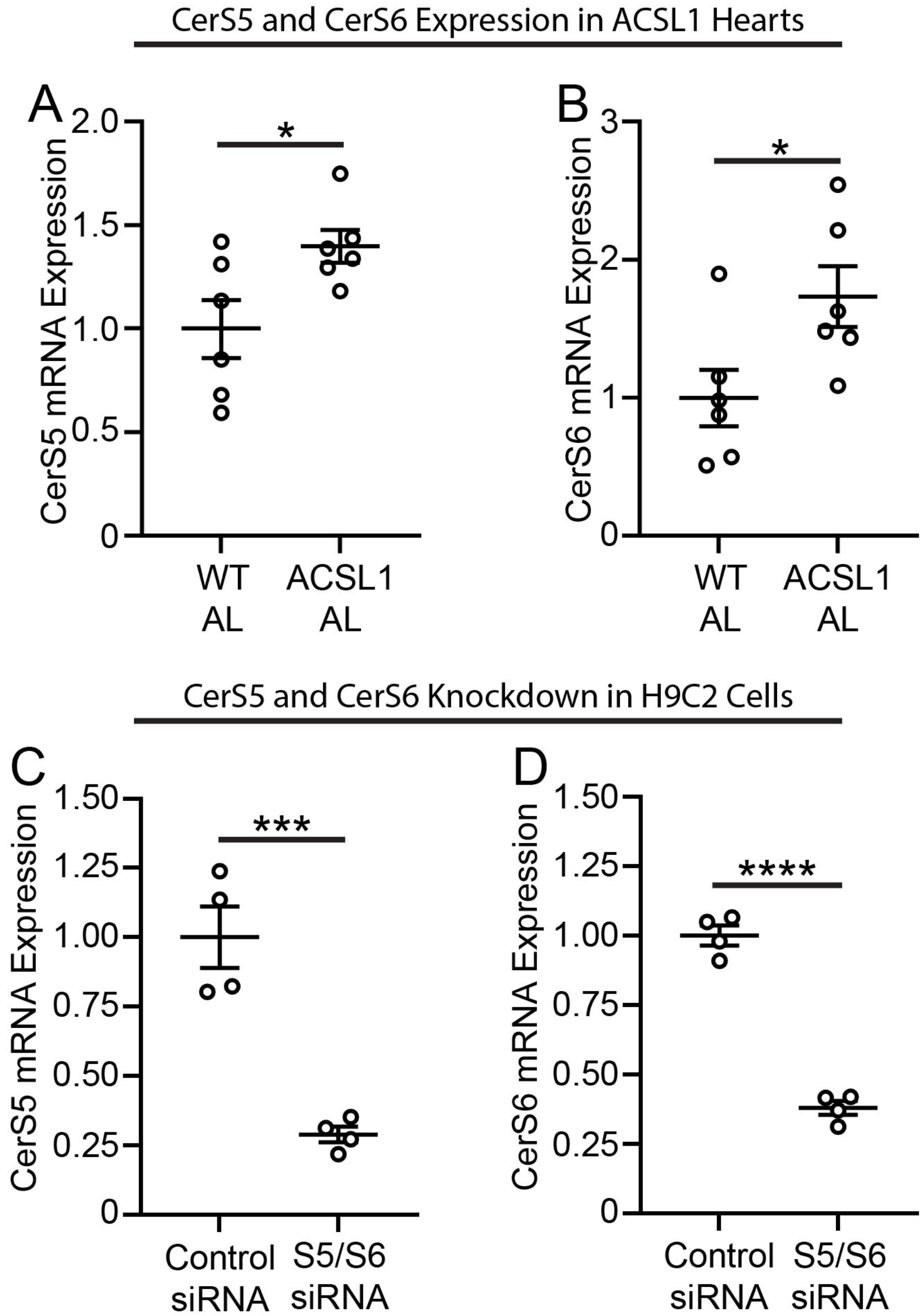

Supplemental Figure S4. Ceramide synthase 5 and 6 mRNA expression in the hearts of MHC-ACSL1 transgenic mice, and ceramide synthase expression in H9C2 cells following siRNA targeting. (A-B) Expression of ceramide synthase 5 (CerS5) and ceramide synthase 6 (CerS6) as assessed by quantitative RT-PCR in 6-week-old wild type and MHC-ACSL1 hearts. All mice are on ad-libitum diets (AL). **(C-D)** CerS5 and CerS6 mRNA levels in rat H9C2 cells 48 hours after treatment with siRNA against rat CerS5 and CerS6 (S5/S6), as compared to cells treated with non-targeting control siRNA. Statistical comparisons in A-D are by unpaired t-test. * $p<0.05$, *** $p<0.001$, **** $p<0.0001$.

Figure S5

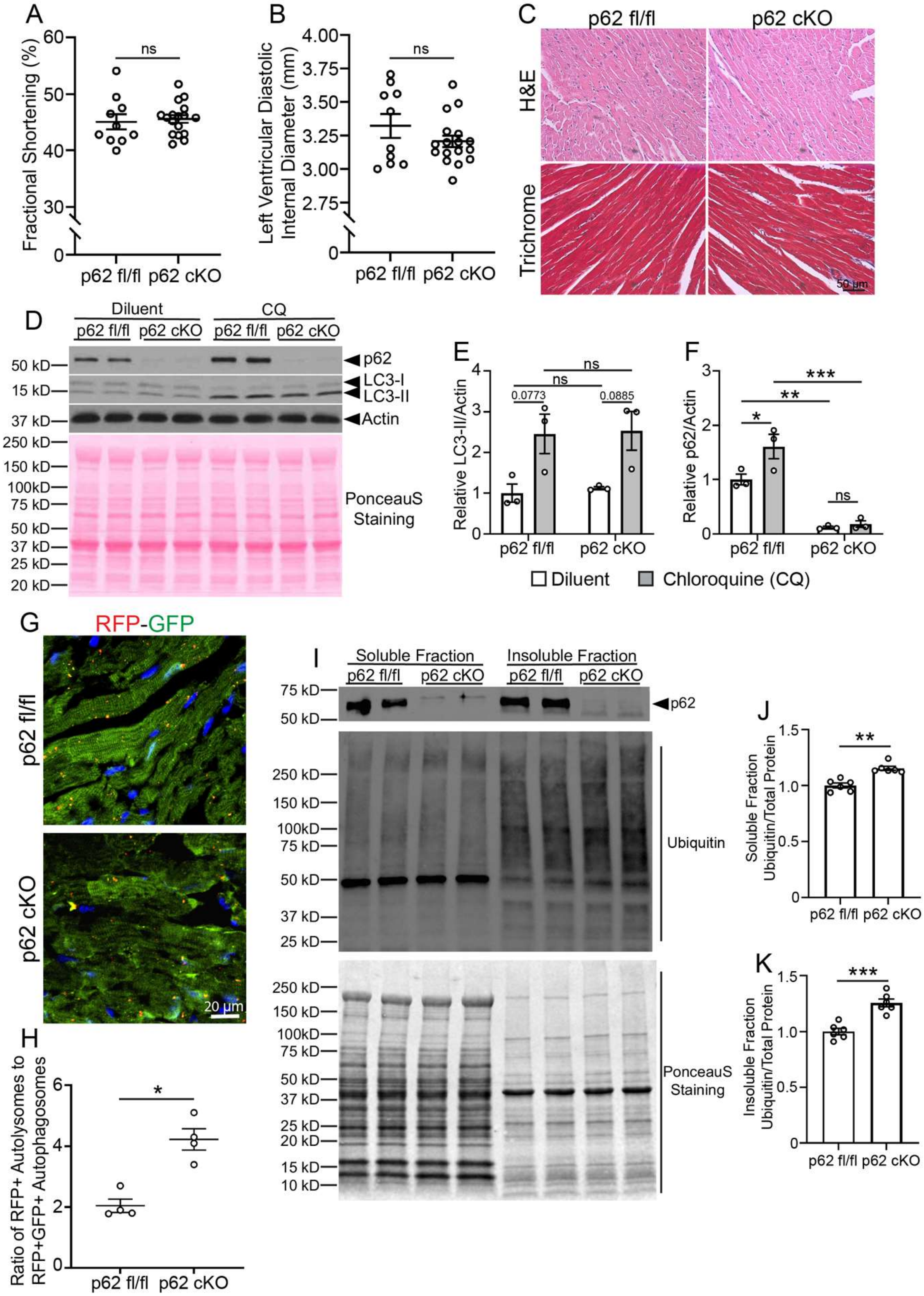
A
Fractional Shortening (%)
ns
p62 fl/fl
p62 cKO
B
Left Ventricular Diastolic Internal Diameter (mm)
ns
p62 fl/fl
p62 cKO
C
p62 fl/fl
p62 cKO
H&E
Trichrome
50 µm
D
Diluent
CQ
p62 fl/fl
p62 cKO
p62 fl/fl
p62 cKO
p62
LC3-I
LC3-II
Actin
PonceauS Staining
E
Relative LC3-II/Actin
0.0773
0.0885
ns
ns
F
Relative p62/Actin
*
**
***
ns
Diluent
Chloroquine (CQ)
G
RFP-GFP
p62 fl/fl
p62 cKO
20 µm
H
Ratio of RFP+ Autolysomes to RFP+GFP+ Autophagosomes
*
I
Soluble Fraction
Insoluble Fraction
p62 fl/fl
p62 cKO
p62
Ubiquitin
PonceauS Staining
J
Soluble Fraction Ubiquitin/Total Protein
**
K
Insoluble Fraction Ubiquitin/Total Protein
***

Supplementary Figure S5. Cardiac myocyte specific p62 ablation does not impair cardiac structure, function, or autophagic flux in young mice. (A-B) %Left ventricular endocardial fractional shortening (A) and left ventricular diastolic internal diameter (B) in 8-week-old Myh6-Cre p62 flox/flox (p62 cKO) or in p62 flox/flox (p62 fl/fl) control mice. Statistical testing by t-test. **(C)** Hematoxylin and eosin (H&E) and Masson's trichrome staining in 12-week-old p62 fl/fl and p62 cKO hearts. **(D)** Immunoblotting in 8-week-old p62 fl/fl and p62 cKO mice treated with IP chloroquine (CQ) injection (dose-80 mg/kg) or diluent control injection 4 hours prior to sacrifice. **(E-F)** Quantitation of LC3B-II (E) and p62 (F) from panel D, normalized to actin expression. Statistical testing by two-way ANOVA with Tukey's test for multiple comparison testing. **(G)** Fluorescent imaging of RFP and GFP expression from frozen sections from p62 fl/fl and p62 cKO mice carrying the CAG-LC3-RFP-GFP transgenic reporter allele. **(H)** Quantitation of ratio of RFP+ autolysosomes to RFP+ GFP+ autophagosomes per high power field from panel G. Puncta were quantified from five separate images per sample and then averaged. Statistical comparison by t-test. **(I)** Immunoblotting of the NP-40 soluble and NP-40 insoluble fractions from 8-week-old p62 fl/fl or p62 cKO hearts. **(J-K)** Quantitation of ubiquitin levels in the soluble (J) and insoluble (K) fractions from panel I. Ubiquitin levels are normalized to Ponceau S staining. Statistical comparison by t-test. For statistical comparisons in this figure, ns $p>0.05$, ** $p<0.01$, *** $p<0.001$.

Figure S6

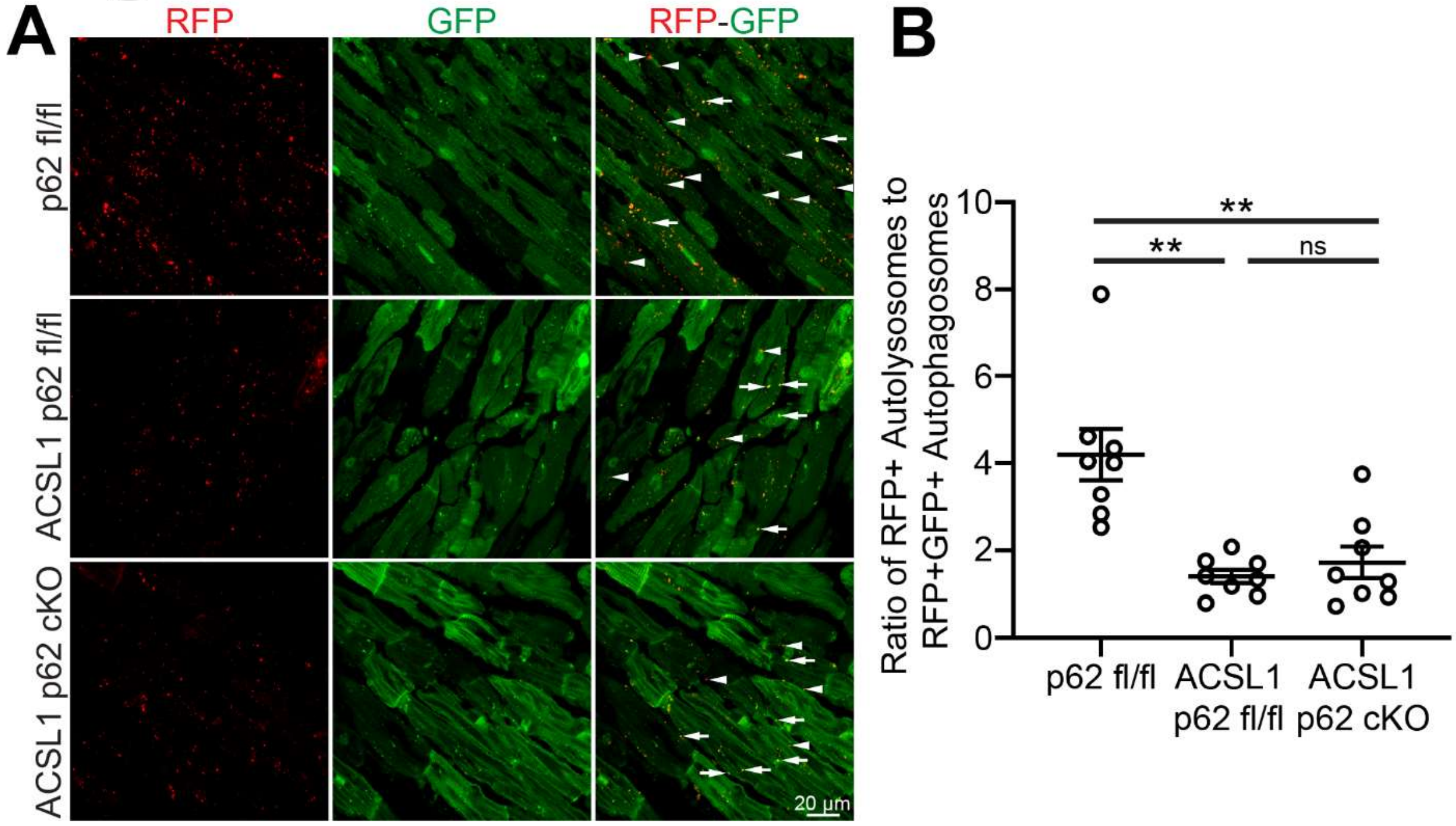

Supplemental Figure S6. Deletion of p62 in cardiomyocytes does not alter the impairment of autophagic flux in cardiomyocytes in ACSL1 transgenic mice. (A) Fluorescent imaging of RFP and GFP expression from frozen sections from 10-week-old hearts from p62 fl/fl vs. MHC-ACSL1 p62 fl/fl vs. MHC-ACSL1 p62 cKO mice. All mice were heterozygous for the CAG-LC3-RFP-GFP transgenic reporter allele. White arrowheads indicate RFP+ GFP – autolysosomes; white arrows indicate double RFP+ GFP+ autophagosomes. **(B)** Ratio of RFP+ GFP- puncta (autolysosomes) to RFP+GFP+ puncta (autophagosomes) from Panel A. Puncta were scored from five images per sample and then averaged. Statistic comparison by one-way ANOVA with Tukey's test for multiple comparison testing. For statistical comparisons in this figure, ns $p>0.05$, ** $p<0.01$.

Figure S7

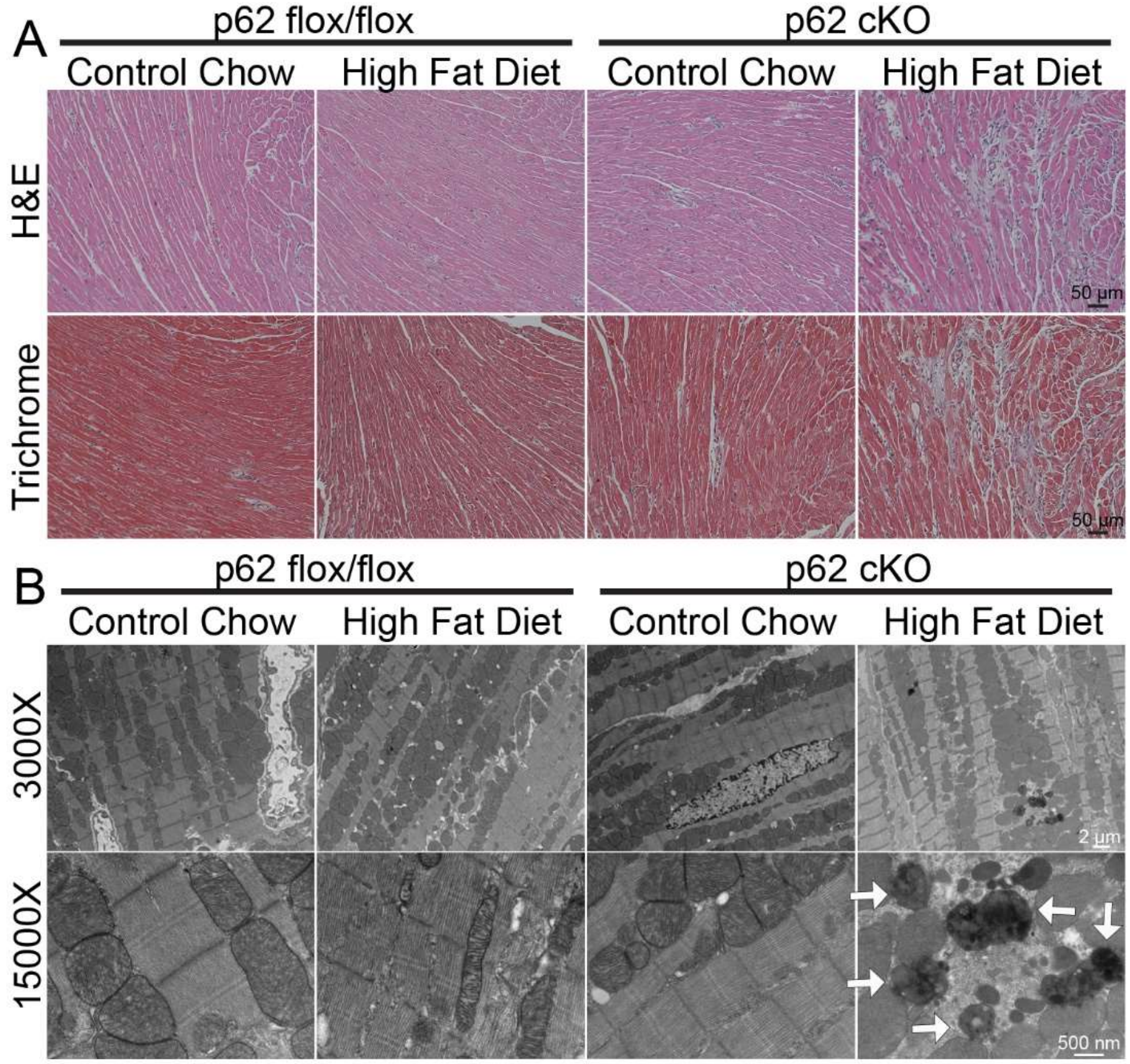

Supplementary Figure S7. Cardiac myocyte ablation of p62 induces fibrosis and protein aggregates in mice fed a high-fat diet. (A) Representative hematoxylin and eosin-stained myocardial sections from 32 week-old p62 fl/fl or p62 cKO mice fed 24 weeks of control chow ad-lib (Chow) or a high-fat diet ad-lib diet (HFD). **(B)** Representative transmission electron micrographs depicting myocardial ultrastructure from mice treated as in A. Arrows point to large electron dense protein aggregates.

Supplemental Table S1: Morphometric data for WT or MHC-ACSL1 mice on indicated diets at 12 weeks of age.

	**Wild-Type Mice Ad-lib Diet (n=24)**	**MHC-ACSL1 Mice Ad-lib Diet (n=25)**	**MHC-ACSL1 Mice IF Diet (n=17)**
**BW (g)**	21.50 ± 0.36	21.77 ± 0.45	20.58 ± 0.46
**HW/BW (mg/g)**	4.85 ± 0.05	6.66 ± 0.23 ****	5.30 ± 0.09 * $^{###}$
**HW/TL (g/mm)**	6.26 ± 0.13	8.77 ± 0.32 ****	6.66 ± 0.14 $^{####}$

Data represent mean ± SEM. Statistical testing by one-way ANOVA followed by post-hoc Tukey's test (for BW) or by non-parametric Kruskal-Wallis test followed by post-hoc testing with Dunn's test (for HW/BW, HW/TL). * $p<0.05$, **** $p<0.0001$ for comparison vs. Wild-type Ad-lib Diet group; $^{###}$ $p<0.001$, $^{####}$ $p<0.0001$ for comparison vs. MHC-ACSL1 Ad-lib diet group. All other statistical comparisons did not meet significance ($p>0.05$). BW = Body Weight, HW = Heart Weight, TL = Tibia Length.

Supplemental Table S2: Echocardiographic data for WT or MHC-ACSL1 mice on indicated diets at 12 weeks of age.

	**Wild-Type Mice Ad-lib Diet (n=4)**	**MHC-ACSL1 Mice Ad-lib Diet (n=9)**	**MHC-ACSL1 Mice IF Diet (n=11)**
**LVIDd (mm)**	3.39 ± 0.08	4.19 ± 0.14 *	3.45 ± 0.06 ##
**LVIDs (mm)**	1.95 ± 0.05	3.39 ± 0.18 ***	2.31 ± 0.10 ###
**FS (%)**	42.33 ± 0.86	19.26 ± 1.86 ****	33.86 ± 1.69 ###
**LV mass (g)**	130.96 ± 6.84	147.62 ± 4.30	113.10 ± 5.66 ##
**HR (beats per min)**	646.4 ± 14.1	553.9 ± 7.2 ***	546.4 ± 5.8 ****

Data represent mean ± SEM. Statistical testing by one-way ANOVA followed by post-hoc Tukey's test (for LVIDs, FS, LV Mass, HR) or by non-parametric Kruskal-Wallis test followed by post-hoc testing with Dunn's test (for LVIDd). * $p<0.05$, *** $p<0.001$, **** $p<0.0001$ for comparison vs. Wild-Type Ad-lib Diet group; ## $p<0.01$, ### $p<0.001$ for comparison vs. MHC-ACSL1 Ad-lib diet group. All other statistical comparisons did not meet significance ($p>0.05$). LVIDd = Left Ventricular Internal Diameter End Diastole, LVIDs = Left Ventricular Internal Diameter End Systole, FS = Fractional Shortening, LV mass = Left-Ventricular mass based on M-mode echocardiography measurements, HR = Heart rate.

Supplemental Table S3: Morphometric data for mice of indicated genotypes at 6 weeks of age.

	**p62 fl/fl mice (n=10)**	**MHC-ACSL1 p62 fl/fl mice (n=10)**	**MHC-ACSL1 p62 cKO mice (n=8)**
**BW (g)**	17.75 ± 0.39	17.88 ± 0.41	18.38 ± 0.58
**HW/BW (mg/g)**	4.79 ± 0.10	6.18 ± 0.10 ****	6.27 ± 0.09 ***
**HW/TL (g/mm)**	5.60 ± 0.19	7.25 ± 0.19 ****	7.54 ± 0.22 ****

Data represent mean ± SEM. Statistical testing by one-way ANOVA followed by post-hoc Tukey's test (for BW, HW/TL) or by non-parametric Kruskal-Wallis test followed by post-hoc testing with Dunn's test (for HW/BW). *** $p<0.001$, **** $p<0.0001$ for comparison vs. p62 fl/fl group; there are no statistically significant differences between MHC-ACSL1 p62 fl/fl and MHC-ACSL1 p62 cKO groups. All other statistical comparisons did not meet significance ($p>0.05$). BW = Body Weight, HW = Heart Weight, TL = Tibia Length.

Supplemental Table S4: Echocardiographic data for mice of indicated genotypes at 6 weeks of age.

	**p62 fl/fl mice (n=8)**	**MHC-ACSL1 p62 fl/fl mice (n=8)**	**MHC-ACSL1 p62 cKO mice (n=8)**
**LVIDd (mm)**	3.21 ± 0.08	3.45 ± 0.05 *	3.41 ± 0.04
**LVIDs (mm)**	1.86 ± 0.07	2.27 ± 0.5 ***	2.26 ± 0.04 ***
**FS (%)**	41.96 ± 1.40	34.35 ± 0.66 ***	33.93 ± 0.90 ***
**LV mass (g)**	68.71 ± 2.24	85.70 ± 3.03 ***	81.37 ± 1.34 **
**HR (beats per min)**	587.9 ± 14.6	547.6 ± 5.9 *	551.7 ± 7.3

Data represent mean ± SEM. Statistical testing by one-way ANOVA followed by post-hoc Tukey's test. * $p<0.05$, ** $p<0.01$, *** $p<0.001$ for comparison vs. p62 fl/fl mice group. All other statistical comparisons did not meet significance ($p>0.05$); there are no statistically significant differences between MHC-ACSL1 p62 fl/fl and MHC-ACSL1 p62 cKO groups. LVIDd = Left Ventricular Internal Diameter End Diastole, LVIDs = Left Ventricular Internal Diameter End Systole, FS = Fractional Shortening, LV mass = Left-Ventricular mass based on M-mode echocardiography measurements, HR = Heart rate.

Supplemental Table S5: Morphometric data for mice of indicated genotypes at 10 weeks of age.

	**MHC-ACSL1 p62 fl/fl mice (n=11)**	**MHC-ACSL1 p62 cKO mice (n=11)**
**BW (g)**	19.48 ± 0.69	20.51 ± 0.67
**HW/BW (mg/g)**	6.48 ± 0.17	6.16 ± 0.12
**HW/TL (g/mm)**	8.43 ± 0.32	7.91 ± 0.26

Data represent mean ± SEM. Statistical testing by unpaired t-test. All statistical comparisons did not meet significance ($p>0.05$). BW = Body Weight, HW = Heart Weight, TL = Tibia Length.

Supplemental Table S6: Echocardiographic data for mice of indicated genotypes 10 weeks of age.

	**MHC-ACSL1 p62 fl/fl mice (n=4)**	**MHC-ACSL1 p62 cKO mice (n=6)**
**LVIDd (mm)**	3.68 ± 0.17	4.78 ± 0.11 *
**LVIDs (mm)**	2.81 ± 0.14	3.86 ± 0.17 **
**FS (%)**	23.53 ± 0.14	12.83 ± 1.93 *
**LV mass (g)**	104.20 ± 6.72	125.30 ± 5.3 *
**HR (beats per min)**	556.2 ± 4.3	536.9 ± 25.37

Data represent mean ± SEM. Statistical testing by unpaired t-test. * $p<0.05$, ** $p<0.01$ for comparison vs. MHC-ACSL1 p62 fl/fl group. All other statistical comparisons did not meet significance ($p>0.05$). LVIDd = Left Ventricular Internal Diameter End Diastole, LVIDs = Left Ventricular Internal Diameter End Systole, FS = Fractional Shortening, LV mass = Left-Ventricular mass based on M-mode echocardiography measurements, HR = Heart rate.

Supplemental Table S7: Echocardiographic data for MHC-ACSL1 p62 cKO mice on indicated diets at 10 weeks of age.

	**MHC-ACSL1 p62 cKO Ad Lib Diet (n=7)**	**MHC-ACSL1 p62 cKO IF Diet (n=7)**
**LVIDd (mm)**	3.96 ± 0.10	3.41 ± 0.10 ***
**LVIDs (mm)**	3.32 ± 0.13	2.29 ± 0.08 ****
**FS (%)**	16.39 ± 1.19	37.47 ± 1.06 ****
**LV mass (g)**	94.20 ± 4.43	67.84 ± 3.07 ***
**HR (beats per min)**	541.6 ± 4.2	585.3 ± 9.5 ***

Data represent mean ± SEM. Statistical testing by unpaired t-test (for LVIDs, FS) and by Mann-Whitney test (for LVIDd, LV Mass, HR). *** $p<0.001$, **** $p<0.0001$ for comparison vs. MHC-ACSL1 cKO Ad Lib diet group. LVIDd = Left Ventricular Internal Diameter End Diastole, LVIDs = Left Ventricular Internal Diameter End Systole, FS = Fractional Shortening, LV mass = Left-Ventricular mass based on M-mode echocardiography measurements, HR = Heart rate.

Supplemental Table S8: Echocardiographic data for MHC-ACSL1 p62 fl/fl vs MHC-ACSL1 p62 cKO mice on intermittent fasting at 28 weeks of age.

	**MHC-ACSL1 p62 fl/fl IF Diet (n=8)**	**MHC-ACSL1 p62 cKO IF Diet (n=6)**
**LVIDd (mm)**	3.66 ± 0.06	4.35 ± 0.17 ***
**LVIDs (mm)**	2.58 ± 0.08	3.71 ± 0.22 ***
**FS (%)**	29.63 ± 1.29	15.11 ± 1.78 ****
**LV mass (g)**	89.80 ± 4.65	105.17 ± 2.94
**HR (beats per min)**	566.0 ± 11.7	550.5 ± 13.7

Data represent mean ± SEM. Statistical testing by unpaired t-test (LVIDd, LVIDs, FS, LV Mass) and by Mann-Whitney test (HR). *** $p<0.001$, **** $p<0.0001$ for comparison vs. MHC-ACSL1 p62 fl/fl IF diet group. All other statistical comparisons did not meet significance ($p>0.05$). LVIDd = Left Ventricular Internal Diameter End Diastole, LVIDs = Left Ventricular Internal Diameter End Systole, FS = Fractional Shortening, LV mass = Left-Ventricular mass based on M-mode echocardiography measurements, HR = Heart rate.

Supplemental Table S9: Morphometric data for p62 fl/fl or p62 cKO mice at 32 weeks of age following 24 weeks of high-fat diet or chow control diet.

	**p62 fl/fl**<br>**Chow Diet (n=7)**	**p62 fl/fl**<br>**HFD (n=7)**	**p62 cKO**<br>**Chow Diet (n=7)**	**p62 cKO**<br>**HFD (n=5)**
**BW (g)**	25.40 ± 0.75	39.08 ± 2.60**	25.9 ± 1.05	38.12 ± 3.56$^{\#\#}$
**BW/TL (g/mm)**	1.48 ± 0.04	2.26 ± 0.15**	1.52 ± 0.07	2.24 ± 0.22$^{\#\#}$
**HW/TL (mg/mm)**	6.69 ± 0.19	7.78 ± 0.42*	6.63 ± 0.21	8.11 ± 0.37$^{\#}$
**LW/TL (mg/mm)**	8.80 ± 0.31	10.14 ± 1.11	8.37 ± 0.23	11.09 ± 1.42

Data represent mean ± SEM. Statistical testing by two-way ANOVA followed by post-hoc Tukey's test. * $p<0.05$, ** $p<0.01$ for comparison vs. p62 fl/fl Chow diet group; $^{\#}$ $p<0.05$, $^{\#\#}$ $p<0.01$ vs. p62 cKO Chow diet group. All other statistical comparisons did not meet significance ($p>0.05$). BW = Body Weight, HW = Heart Weight, LW = Lung weight, TL = Tibia Length, HFD = High-fat diet.

Supplemental Table S10: Echocardiographic data for p62 fl/fl or p62 cKO mice at 32 weeks of age following 24 weeks of high-fat diet or chow control diet.

	**p62 fl/fl Chow Diet (n=7)**	**p62 fl/fl HFD (n=7)**	**p62 cKO Chow Diet (n=7)**	**p62 cKO HFD (n=6)**
**LVIDd (mm)**	3.39 ± 0.10	3.49 ± 0.13	3.53 ± 0.08	4.02 ± 0.28
**LVIDs (mm)**	1.97 ± 0.11	1.91 ± 0.08	2.18 ± 0.07	3.03 ± 0.40 ** # ‡‡
**FS (%)**	42.23 ± 1.84	45.14 ± 1.18	38.27 ± 0.79	26.2 ± 4.02 *** ##### ‡‡
**LV mass (g)**	84.57 ± 4.61	96.114 ± 7.0	86.90 ± 3.50	100.71 ± 4.98 *
**HR (beats per min)**	591 ± 15.5	621 ± 14.2	650 ± 10.0	604 ± 26.6

Data represent mean ± SEM. Statistical testing by two-way ANOVA followed by post-hoc Tukey's test. * $p<0.05$, ** $p<0.01$, *** $p<0.001$ for comparison vs. p62 fl/fl Chow diet group; # $p<0.05$, #### $p<0.0001$ for comparison vs. p62 fl/fl HFD group; ‡‡ $p<0.01$ for comparison vs. p62 cKO Chow diet group. All other statistical comparisons did not meet significance ($p>0.05$). LVIDd = Left Ventricular Internal Diameter End Diastole, LVIDs = Left Ventricular Internal Diameter End Systole, FS = Fractional Shortening, LV mass = Left-Ventricular mass based on M-mode echocardiography measurements, HR = Heart rate.

Supplementary Table S11: Characteristics of individuals for data reported in biochemical analysis of human ventricular myocardium (in Figure 8A-C).

	Non-Diabetic Control (n=3)	Diabetic (n=3)	P-value
**Age**	52.0 ± 2.8	66.7 ± 3.3	0.051
**Diabetes (n)**	0	3	N/A
**Insulin Use (n)**	0	3	N/A
**LV Mass (g)**	239.7 ± 20.9	396.5 ± 30.9	0.0566
**Heart Weight (g)**	387.7 ± 25.5	683.5 ± 49.9	0.0301
**Body Weight (kg)**	107 ± 17.2	97.0 ± 4.0	0.669
**BSA (m2)**	2.27 ± 0.17	2.15 ± 0.13	0.651
**LVMI (g/m2)**	105.4 ± 1.5	176.5 ± 11.0	0.016
**HMI (g/m2)**	171.3 ± 2.9	304.2 ± 17.48	0.010
**LVEF (%)**	62.5 ± 1.44	63.3 ± 1.36	0.79

Data represent mean ± SEM. Statistical testing is by t-test.

Supplementary Table S12: Characteristics of individuals for data reported in histologic analysis of human ventricular myocardium (Figure 8D-F).

	**Non-Diabetic Control (n=3)**	**Diabetic (n=3)**	**P-value**
**Age**	66.0 ± 3.1	59.3 ±7.3	0.954
**Diabetes (n)**	0	3	N/A
**Body Weight (kg)**	73.3 ± 5.4	93.3 ± 6.8	0.835
**BMI**	24.8 ± 1.9	28.9 ± 0.6	0.995
**LVEF (%)**	67.8 ± 2.23	65.0 ± 2.36	0.79

Data represent mean ± SEM. Statistical testing is by t-test.
